# Feature engineering solution with structured query language analytic functions in detecting electricity frauds using machine learning

**DOI:** 10.1038/s41598-022-07337-7

**Published:** 2022-02-28

**Authors:** Simona-Vasilica Oprea, Adela Bâra

**Affiliations:** Department of Economic Informatics and Cybernetics, Bucharest University of Economic Studies, Romana Square 6, 010374 Bucharest, Romania

**Keywords:** Energy science and technology, Engineering

## Abstract

Detecting fraud related to electricity consumption is usually a difficult challenge as the input datasets are sometimes unreliable due to missing and inconsistent records, faults, misinterpretation of meter reading remarks, status, etc. In this paper, we obtain meaningful insights from fraud detection using real datasets of Tunisian electricity consumption metered by conventional meters. We propose an extensive feature engineering approach using the structured query language (SQL) analytic functions. Furthermore, double merging of datasets reveals more dimensions of the data allowing better detection of irregularities in consumption. We analyze the results of several machine learning (ML) algorithms that manage cases of weakly correlated features and highly unbalanced datasets. The skewness of the target is approached as a regular characteristic of the input data because most of consumers are fair and only a small portion attempt to mislead the utility companies by tampering with metering devices. Our fraud detection solutions consist of combining classifiers with an anomaly detection feature obtained with an unsupervised ML algorithm—Isolation Forest, and extensive feature engineering using SQL analytic functions on large datasets. Several techniques for feature processing enhanced the Area Under the Curve score for Decision Tree algorithm from 0.68 to 0.99.

## Introduction

The majority of Non-Technical Losses (NTL) in power systems are caused by fraud in energy consumption. From the earliest times, detection of fraudulent consumption has been a challenge as it means more costs for grid utilities^1^. In most countries, these costs are included in the final electricity price, therefore, society pays for the fraud of deceiving consumers. In this context, detection is essential. The efforts to catch the energy spillage and costs they generate should be recovered from fraudsters to set an example. Often, deceiving consumers come from poor communities^2^, therefore prevention should also consider social issues and provide more incentives towards fair behavior. Thus, fraud detection in electricity consumption considerably reduces the NTL and discourages similar behavior. However, an efficient machine learning model to identify fraudsters is not easy to obtain^3,4^. The main problem lies in the lack of correlation between data features and target. If the target is less dependent on the input variables, the model has real issues to learn and provide high performance^5^.

One class imbalance is normal in fraud detection. For example, in datasets related to subscription cancelation, cancer diseases, migration of bank clients, fraudulent transactions, energy theft, etc., the target is highly imbalanced. Most of the sample will be in the “0” or no-fraud class and a small minority will be in the “1” or fraud class. Also, in electricity consumption, most consumers are honest, only a minority class alters the consumption records or tampers with metering system. There are different patterns of altering the consumption^6^, but they are not visible with conventional meters that are read once a month or even less often. Therefore, it is more difficult to catch irregularities in electricity consumption measured with conventional meters than with smart meters. Usually, the fraudsters have a malicious behavior and claim a smaller consumption that the real one to pay less than the value for the consumed electricity. Furthermore, the difficulty results from the highly unbalanced datasets that are specific in the case of fraud detection. Usually, the training datasets are either under-sampled or over-sampled or processed using Synthetic Minority Oversampling Technique (SMOTE), which has several variants, such as Borderline-SMOTE, SMOTE-NC, SMOTE SVM, or Adaptive Synthetic Sampling (ADASYN). However, the highly unbalanced target in the test data together with a low feature correlation could lead to accurate solutions, but with low precision or recall that are the most significant factors for classification problems.

Although the usual efforts to improve feature correlation and skewness of the target have been exhaustively performed, the intrinsic nature of the dataset limits the performance of the ML algorithms. As a rule of thumb, even if the algorithms are simpler or if their hyper-parameters are less tuned, the accuracy, precision, recall, AUC score and other significant metrics should be reasonable. Therefore, the remaining problem exists in the feature engineering that is probably the most important step in solving fraud detection problems, especially when there is no evident correlation between features of the datasets and target (target in our case is a normal consumer as 0 or a fraudster as 1).

The problem—fraud detection is a sensitive one because it is not convenient to accuse a consumer of such behavior without solid evidence. Therefore, the scope of the fraud detection analyses in our case is to maximize the *true positive* while minimizing the *false positive* results. However, for utility companies that perform ML analyses, it is better to identify as many suspicious consumers as possible and perform further investigations to clarify if the consumers have acted fraudulently or not. Furthermore, if the available funds for further periodic inspections are limited, then another approach would be to identify less *false positive* results that usually lead to less identified fraudsters. Therefore, in creating and extracting new features, it is important to study the input dataset, to understand the problem, the existing features and their impact on the target.

In many cases, the target data is missing, therefore the problem becomes an unsupervised one and could be solved with anomaly detection algorithms^7^. The difference between logistic regression, decision tree or other classifiers and anomaly detection algorithms is the existence of the target in the training set. In most cases, the utility companies’ suspicions rely on neighbors’ complaints or denouncements and periodic on-site investigations. When a target is present, it is tempting to consider classification, but for a very skewed target, it is recommended to implement anomaly detection as malicious consumption patterns may not repeat, so the supervised ML algorithms are not able to learn from the input data. Therefore, our proposal in electricity consumption fraud detection is to combine two machine learning approaches: anomaly detection, such as the *Isolation Forest* unsupervised algorithm to create a new feature for running classification combined with complex supervised algorithms such as *eXtreme Gradient Booting (XGB), Random Forest (RF)* or *Light Gradient Boosting (LGB)*.

The objective of this paper is to apply an extensive feature engineering with SQL analytics to data variables, creating new meaningful variables and enhance the classification scores. This approach reveals more dimensions of the datasets that are merged twice leading to better performance indicators for several classifiers from which one can choose the best solution for frauds detection. Moreover, new features are created using unsupervised ML algorithms, from anomaly detection, aggregation and derived functions aiming to improve the initial results without feature engineering and decrease the cost of grid operators that have to plan periodic on-site investigations. Therefore, the motivation behind this paper is to improve the metrics that show the accuracy of the classification process and ML algorithms, such as: precision, recall, F1 score, AUC and confusion matrix that are indicative for assessing and comparing the classifiers. A flowchart for the electricity consumption fraud detection solution is provided to describe our research methodology and ensure its replicability.

The current research contributes to the state-of-the-art by proposing extensive feature engineering extracted with SQL analytic functions, aggregation and derived functions. To the best of our knowledge, the SQL analytic functions have not been implemented yet to create new features. In this sense, we propose an extensive feature engineering procedure, modeling the data processing stage and providing examples of feature engineering with SQL analytic functions that capture the irregularities in data and offer solid ground for classification purpose. Additionally, after the datasets have been processed, double merging is applied. Firstly, the master and detail aggregated datasets are joined. Secondly, the resulted dataset is again merged with the detail datasets after applying analytic functions. This approach will transform and generate numerous features that highlight the anomalies and spikes in current and previous invoices. The usual behavior of the fraudster is to tamper with the meter half of the month and normally consume the rest of the month, so that the monthly consumption on average remains the same. However, sometimes, the fraudster forgets to alter the meter or to restore it, generating irregularities in the monthly consumption. Therefore, we propose to add new features obtained with SQL analytic and aggregate functions and, in addition, to investigate the data irregularities with an anomaly detection algorithm that will better highlight anomalous consumer behavior.

This paper is structured in five sections. In the second section, a comprehensive literature survey is presented considering the most recent and relevant scientific research. The third section describes the methodology of the current study, emphasizing the main steps and a formal presentation of the model. The original contribution and novelty of this paper are discussed in “Methodology” section, underlying the procedure of extensive feature engineering with SQL analytic functions, aggregation and double merging of datasets. The novelty also exists due to the combination of two supervised and unsupervised ML algorithms—anomaly detection to create a new feature and a classifier—to improve the performance of the model. The results of simulation are presented in the fourth section. Finally, conclusions are drawn in “Conclusion” section.

## Literature review

Numerous valuable scientific papers have been written on the topic of fraud detection in electricity consumption using several datasets on consumption from various countries such as India^8^, China^1,9^, Spain^10^, Ireland^11^, Brazil^2^, Malaysia^12^ and Uruguay^13^. Most of them concentrate on either classification or anomaly detection algorithms. Classification of the load profile was implemented to detect anomalies in electricity consumption in a village in India^8^. A detection methodology is designed using hierarchical clustering and decision tree classification to find out abnormalities in load patterns. It uses validation profiles to ensure accurate classification between anomalies and theft.

Supervised ML was applied for fraud electricity consumption detection on a dataset from Spain^14,15^ emphasizing the higher performance of the DT, RF and Ada Boost (AB) with Naive Bayes models. In addition, data from Endesa, one of the distribution companies in Spain, was used to analyze and identify NTL^16^. A knowledge-based system was built-up based on the expertise of onsite controls, text mining, neural networks and statistics. Detection of NTL using smart meter data from Endesa and supervised learning^10^ emphasize several classifiers of which XGB was the best performer. In another study, deep neural networks are the best performing algorithms using input data from Endesa (Spain)^17^.

Another approach was noted in Ref.^18^, highlighting new aspects related to frauds in smart grids such as cyberattacks^6^. Prediction was used for detecting energy fraud in smart meter operations of the trial Irish public dataset^19^ using an artificial neural network to identify quasi-normal energy consumption and enable the detection of anomalies. Furthermore, the clustering technique by fast search and find of density peaks were applied in Ref.^13^ using Irish smart metering data to identify the abnormal consumers from their load profiles.

Interesting datasets from China were analyzed from the theft detection point of view with empirical mode decomposition and k-nearest neighbors^9^. A valuable research was provided in Ref.^1^ using the same input data (from State Grid Corporation of China utility company). The authors analyzed smart meter readings, separating the data into two sets for normal consumers and fraudsters. The irregularities were investigated with wide and deep convolutional neural networks. The data was highly correlated with the target and these correlations were also studied separately. An AUC-score of almost 0.8 is reached using two-dimensional electricity consumption data. Neural networks were also used in Ref.^20^ to classify time series and provide an analysis of the models capability and network structures.

Using historical electricity consumption data for three towns in Malaysia, theft was detected with Support Vector Machine (SVM) and fuzzy logic^12,21,22^. The DT and SVM were implemented in Ref.^23^ to detect fraud in electricity consumption in smart grids^24^. A system to detect energy theft was created in Ref.^25^ using ML and statistics. Three stages of decision-making to detect theft were considered. Large datasets from Uruguay were analyzed using a Bayesian risk framework that assists the utility company to perform on-site inspections and plan the budget for NTL^13^. The results proved that the pipeline approach can be adapted to other datasets.

Most algorithms that are used in electricity theft are supervised, but unsupervised and hybrid algorithms are gaining increasing importance as well^26–29^. Furthermore, a combination of supervised algorithms (classifiers) and unsupervised algorithms (especially clustering) is also possible^30,31^. Model selection is an important step emphasized in many scientific research paper, such as^32^ that handles imbalanced data and focuses on a model dynamic selection, or Refs.^33,34^ that describe several solutions for detection of NTL in the electricity grid.

Our proposed approach can be implemented for similar datasets measured by conventional meters, but the SQL analytic functions are appropriate for time series generated by smart meters as well. For smart meter-recorded data, there are other approaches that involve specific machine leaning algorithms and time series feature extraction library for electricity consumption fraud detection in smart grids^7^. However, although the trend is to gradually replace the conventional meters, there are still large regions even in the developed countries that continue to measure electricity with conventional meters. So, the conventional meters are far from being replaced in the next ten years, both meter types (conventional and smart) will coexist for a longer period of time and will require performant and practical methods to detect frauds^35^.

In Table 1, the main characteristics of other scientific research papers on the electricity consumption fraud detection are provided.

**Table 1:** Characteristics of other scientific research papers on the electricity consumption fraud detection.

Ref.	Data provider	Meter type	Algorithm	Skewness	Data processing	Feature engineering
^1^	State Grid Corporation of China	Smart meter	Three-sigma Rule, LR, RF, SVM, wide and deep convolutional neural network (CNN)	Not approached	Interpolation max–min scaling	No
^2^	Brazilian electric utility	Smart meter	Optimum-path forest classifier	Not approached	Feature selection with black hole algorithm	No
^4^	Brazilian electric utility, CPFL Energia	Smart meter	CNN	Not approached	Time series and image processing	Image feature extractors
^5^	Irish Commission for Energy Regulation (CER)	Smart meter	RF, KNN, SVM, NN, gradient boosting machine (GBM) classification model	Not necessary as there is no fraud indication	Finite mixture model clustering for customer segmentation	Genetic programming algorithm
^6^	Irish CER	Smart meter	SVM	Not necessary	Clustering	No
^7^	Irish CER	Smart meter	Spectral residual-convolutional neural network and an anomaly trained model based on martingales	Not necessary	Data transformation, feature extraction, Fisher discriminant analysis	No
^8^	Indian utility company, Gujarat Urja Vikas Nigam Limited	Smart meter	Data mining techniques: hierarchical clustering and DT	Solved using generative adversarial model	Filling missing values with ARIMA and exponential smoothing, data filtering with Savizky Golay	No
^9^	State Grid Corporation of China	Smart meter	KNN	ADASYN	Interpolation, empirical mode decomposition	Time series feature extraction
^10^	Spanish utility company, Endesa	Smart meter	SVM, LR, KNN, XGB	Undersampling	Data cleaning, feature extraction from different sources	No
^11^	Irish CER	Smart meter	Two-level anomaly detection framework based on regression DT, reduced error pruning tree, DT, LR, RF, ANN, SVM	Not necessary	Outliers, feature extraction from initial variables	No
^12^	Tenaga Nasional Berhad Distribution-TNBD, Malaysia	Conventional meter	SVM	Weighted algorithm to balance the sample ratio	Data mining, normalization feature extraction, load profile	No
^13^	Uruguayan utility (UTE)	Conventional meter	SVM, RF, ANN	Handled by the Bayes minimum error approach, synthetic frauds are simulated over real consumption	Not mentioned	No
^14^	Spain, gas natural Fenosa distributing electricity, gas	Conventional and smart meters	Naive Bayes, KNN, DT, RF, ANN, SVM, Gradient descent Decision Tree, AB	Not mentioned	Load profiles, fraud score	Feature construction, odd ratio
^15^	Naturgy, energy providers in Spain	Smart meters	XGB, LGB, CB	Under- and Over-representation campaigns	Data cleaning and extraction, profiling variables	No
^16^	Endesa, Spain	Conventional meters	Knowledge-based system, detection rules	Not mentioned	Checking the coherence, normalization, load pattern	Text mining
^17^	Endesa, Spain	Smart meters	Hybrid neural network, long short-term memory (LSTM), Multi-layer perceptron (MLP), SVM, LR, RF, CNN	Metrics assess the ranking of samples	Data imputation, weekly profiles	No
^18^	Irish CER	Smart meter	ANN (MLP)	Not necessary	Data cleaning, normalization, feature extraction	No
^20^	44 benchmark datasets	Smart meter	Time series based on a bag-of-features, fully convolutional network, MLP, residual networks (ResNet)	Not mentioned	PCA to reduce dimensionality	No
^21^	TNBD, Malaysia	Not specified	SVM, fuzzy logic, if–then rules	Not mentioned	Load patterns, decision-making system using SQL	No
^22^	India	Smart meters	SVM	Not mentioned	Load profiles and segmentation, noise free data	No
^23^	USA	Aggregated data	DT, SVM, hybrid	Not mentioned	Data conversion, normalization	No
^24^	Irish CER	Smart meters	Anomaly detection framework, linear programming	Not necessary	Data cleaning, calculate discrepancy	No
^25^	Singapore	Smart meters	MLP, recurrent neural network (RNN), LSTM, gated recurrent unit (GRU)	Not mentioned	Time series, accumulative data	No
^26^	Brazilian electrical power company	Load profiles	OPF, K-means, Birch, affinity propagation (AP), and Gaussian mixture model, SVM	Set of parameters that maximized the accuracy	Optimum-path forest (OPF) clustering algorithm	Multivariate Gaussian distribution
^27^	Power distribution companies in the Czech Republic	Smart meters	Data clustering	Not mentioned	Load segmentation, association rule mining, most frequent itemsets filtering	No
^29^	Irish CER	Smart meters	SVM, non-linear non-convex optimization problem solved with semi-definite programming relaxation	Not necessary	Breakout detection is used for extracting features from consumption time series	No
^31^	Irish CER	Smart meters	Gustafson-Kessel fuzzy clustering algorithm, K-means and GMM; DBSCAN clustering and SVM	Not necessary	Feature extraction for irregular consumption detection	No
^32^	UCI and KEEL repositories, 24 imbalanced datasets	Smart meters	Ensemble classification, dynamic selection of classification models	Data partition hybrid sampling, boundary minority class weighted over-sampling (BMW-SMOTE)	Not mentioned	No
^35^	Tunisian Company of Electricity and Gas (STEG)	Conventional meters	AB, DT, KNN, LGB, MLP, QDA, RF, LR, SGD, XGB	SMOTE	Data conversion, encoding, merging	Aggregation functions, multivariate Gaussian distribution
^53^	State Grid Corporation of China	Smart meters	CNN, LSTM	SMOTE	Data imputation, load patterns	No

These studies can be compared by the meter type, algorithms, skewness treatment, data processing and feature engineering characteristics. From Table 1, we notice that out of the 30 references that comprehensively studied fraud detection, only 5 are related to the conventional meter-recorded data and only 7 propose feature engineering.

## Methodology

**Figure 1:**
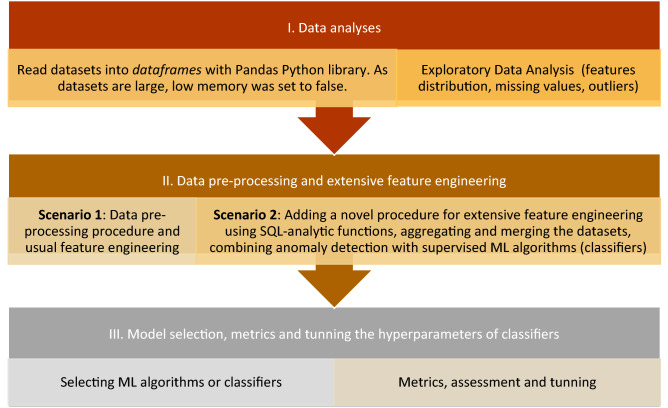
Methodology steps.

The methodology steps are shown in Fig. 1. These steps are: data analyses, pre-processing, feature engineering, model selection, metrics and tuning. In the first step (I. Data analyses), the datasets about consumers and invoices are read into Pandas *dataframes* and Exploratory Data Analysis (EDA) are performed focusing on Gaussian distribution, missing values, outliers, etc. EDA is carried out to extract and understand valuable information from large datasets. This stage is significant and might reveal issues in data, such as missing data, skewness, outliers, etc. In the second step (II. Data pre-processing and extensive feature engineering), we propose two scenarios. The first includes the usual data pre-processing and feature engineering. Scenario 1 derives from the first step and consists of a classic data pre-processing procedure and feature processing. In the Scenario 1, outliers, non-numerical and missing values are handled, whereas in Scenario 2, an extensive calculation is performed using SQL analytic functions and aggregation, joining the datasets, and inserting features that reflect the anomaly in data. Scenario 2 is a novel approach aiming to create more features to enhance the results. It consists of a procedure that processes the datasets considering their type: master (consumers data) and detail (invoice data). In essence, we create two more dimensions by adding new features:

1. with SQL analytic functions that identify the differences between invoices (creating the first new dimension of the consumption data);
2. with aggregate functions that reduce the initial detail dataset (invoices) with group functions (creating the second new dimension of the consumption data).

Furthermore, the aggregated detail dataset is merged with the master dataset (with consumers’ characteristics) and the result is merged with the first new dimension created with SQL analytic functions. The purpose of these transformations and double merging is to identify irregularities in the consumption patterns and improve the performance of the model. A new feature is created with an unsupervised algorithm to better identify anomalies in consumption.

Scenario 1 is a regular stage that is usually performed by researchers, but we propose and test the case with and without Scenario 2 and draw the conclusion that by creating new features the results are significantly improved.

Running the ML algorithms, assessing the results and tuning the models are included into the third step (III. Model selection, metrics and tunning of the hyperparameters of classifiers). Therefore, in the third step, we run several ML algorithms in both scenarios from previous step to classify the consumers into normal or potential fraudsters, tune the parameters of the algorithms (known as hyperparameters) and select the algorithm that provides the best results considering the usual metrics for classification such as F1 score, ROC-AUC and confusion matrix. More details regarding the methodology steps are offered in the following sections.

Therefore, the novelty and contribution of our approach consist of:

- analyzing real datasets measured by conventional meters that represent a challenge for grid operators due to the major issues such as missing data, encoding, skewness, etc. and providing reliable results for a fraud detection classification problem following the above methodology steps;
- proposing a new stage (Scenario 2) in the data pre-processing and extensive feature engineering step that computes more features aiming to improve the results of the classification problem. This stage is in a form of a procedure for extensive feature engineering using SQL-analytic functions, aggregation, merging the datasets and combining anomaly detection with supervised ML algorithms;
- providing a salient tool for grid operators to identify the suspicions consumers that have to be further investigated. With performant classification, the costs related to periodic on-site investigations and non-technical losses are therefore reduced.

### Data analyses

Let us define ${df}_{i}^{type}({m}_{i},{f}_{i})$ as datasets for fraud detection purpose.

- ${m}_{i}$—dataset samples, ${m}_{i}\in [{m}_{1},\dots ,{m}_{s}]$, $s$—number of samples;
- ${f}_{i}$—dataset features, ${f}_{i}\in \left\{{f}_{1},\dots , {f}_{n}\right\}$, $n$—number of features;
- $type$—type of dataset, $type\in \left\{master, detail\right\}$.

Usually, there is one master dataset with the characteristics of the electricity consumers, including the target, and one or several datasets with details regarding consumers’ invoices, readings and status of the meters. The datasets are read and analyzed by performing EDA, checking the feature distribution, missing values, outliers, etc. This step is usually essential for data processing and requires a deep understanding of the field.

### Data pre-processing and extensive feature engineering

At this step, two procedures are proposed. The first one corresponds to scenario 1 and consists of data pre-processing or changing the features of the analyzed datasets, regardless of their type. It contains the usual pre-processing operations, such as:

- data type conversions (for instance, from string to date or to number);
- computation of new features (extracting valuable information from existing features, obtaining so called delta features, i.e., delta time that is a difference of time or delta index that is a difference of consumption);
- encoding and mapping some seldom values to the most frequent ones, correcting the inconsistent data and eliminating missing values to allow ML algorithms to run.

Therefore, we define a procedure, $feature\_change \left({df}_{i}^{type}\right)$, that returns the pre-processed datasets ${pp\_df}_{i}^{type}$.

1 $$featur{e}\_{change}\left({df}_{i}^{type}\right)\to {p{p}\_{df}}_{i}^{type}.$$

In addition to the first scenario, we design a procedure, $feature\_engineering$, that corresponds to scenario 2 designed for more advanced processing of the datasets. It considerably increases the performance of the ML algorithms as it combines the power of two types of algorithms: unsupervised and supervised. Furthermore, the complex $feature\_engineering$ procedure is based on SQL analytic functions, aggregation and double merging of the processed datasets into a dataset $df$ containing multiple dimensions of the consumption data. The parameters of $feature\_engineering$ are the processed datasets explained in Eqs. (3)–(11).

2 $$featur{e}\_{engineering\left(*\right)}\to df.$$

Analytic functions implemented in SQL are applied to the detail datasets. They return an aggregate result based on a group or a range of records and differ from the aggregate functions, mentioned below, as they may return multiple results for each group by creating specific partitions. The group of records is defined as a window using an analytic clause ($analyti{c}_{clause}$). Therefore, for each record, a sliding window of records is created. The sliding window defines a range of records to perform the calculations starting from a current record. The window size is either a physical number of records or a logical interval^36^. Using analytic functions on numerical features ${f}_{i\_num}$, the differences between the preceding/following and current invoices are calculated and irregularities or spikes, especially in consumption, are easily detected. The partitions created with $PARTITION \,BY$ allow us to calculate differences inside groups of consumers defined by features (i.e., category, district, tariff type, and so on). Then, $ORDER \,BY$ indicates features (such as invoice date or invoice month) based on which the computation is performed in relation to a ${window}_{clause}$. Thus, we applied analytic functions ${analytic}_{f}$ to the pre-processed detail datasets ${pp\_df}_{i}^{detail}$ to obtain new features that provide a better image of the invoices and extract more meaningful insights in a new *dataframe* $analytic{\_df}_{i}^{detail}$.

3 $${pp\_df}_{i}^{detail}.{analytic}_{f}\left({f}_{i\_num}\right) OVER \left(analyti{c}_{clause} {window}_{cluase}\right)\to analytic{{}\_{df}}_{i}^{detail},$$

4 $$analyti{c}\_{clause}=PARTITION \,BY \,{f}_{i} \,ORDER\,BY {f}_{i},$$

5 $${window}\_{clause}=\frac{ROWS}{RANGE}\frac{\left\{\frac{preceding}{following}\right\}}{}\left\{current row\right\}.$$

If ${window}_{clause}$ is omitted, then an implicit clause—*range between unbounded preceding and current row* is applied. Some of the analytic functions implemented by most of the database management systems are:

6 $${analytic}_{f}\in \left\{dense\_rank, first, lag, last, lead, max, min, percent\_rank, rank, ratio\_to\_report\right\}.$$

For all ${pp\_df}_{i}^{detail}$, we will reduce dimensionality (samples) by grouping by one or more selected features ${f}_{sel}$ that can be identifiers or specific features and aggregate the other numerical features ${f}_{i\_num}^{{agg}_{f}}$ using several aggregation functions $ag{g}_{f}$. Thus, a reduced dataset $agg{\_df}_{i}^{detail}$ will result. The scope of calculating aggregated features is to compare them with the details and identify anomalies.

7 $${pp\_df}_{i}^{detail}.groupby\left({f}_{sel}\right).agg\left({f}_{i\_num}^{{agg}_{f}}\right)\to agg{\_df}_{i}^{detail},$$

8 $$ag{g}_{f}=\left\{min, mean, median, max, sum, std, var\right\}.$$

Additionally, with the aggregate functions, new features are calculated, such as: ${f}_{i\_num}^{range}$ and ${f}_{i\_num}^{max\_mean}$.

9 $${f}_{i\_num}^{range}={f}_{i\_num}^{max}-{f}_{{i}_{num}}^{min},$$

10 $${f}_{i\_num}^{max\_mean }=\frac{{f}_{i\_num}^{max}}{{f}_{i\_num}^{mean}}.$$

The aggregated datasets $agg{\_df}_{i}^{detail}$ are merged with the pre-processed master dataset ${pp\_df}_{i}^{master}$, and the result is again merged with the analytic detail datasets $analytic{\_df}_{i}^{detail}$, resulting $df$, that is the return of the $feature\_engineering$ procedure. Hence, the aggregated values can be compared with the monthly values, identifying spikes and irregularities that could indicate an anomalous behavior.

11 $$df=join\left({analytic\_df}_{i}^{detail},join\left(agg{\_df}_{i}^{detail},pp\_{df}_{i}^{master} \right)\right).$$

Extracting the target $y$ from $df$ and defining $X$ are the next steps.

12 $$y= df\left(target\right),$$

13 $$X=df-y.$$

The result $X$ will be scaled as the scaling process improves the performance of ML algorithm and increases the convergence speed.

14 $$scale{d}\_{X}=scaler\left(X\right).$$

There are different strategies for scaling, depending on our objectives and the characteristics of the data. The first option is $MinMax scaler$ as it does not distort the data. $Robust \,scaler$ is also indicated when outliers are numerous. Whereas $Standard\, scaler$ and $Normalizer$ are used to standardize or normalize the data.

15 $$scaler\in \left\{MinMax, \,Standard, \,Robust, \,Normalizer\right\}.$$

Then, an anomaly detection algorithm is implemented to identify possible anomalies in the dataset $scaled\_X$ and create a new and valuable feature ${f}^{anomaly}$ that will be added to the dataset to create ${scaled\_X}^{anomaly}$ dataset.

16 $$anomal{y}\_{detection\left(scale{d}\_{X}\right)}\to {f}^{anomaly}\to {scale{d}\_{X}}^{anomaly}.$$

Using SQL analytic functions, Eqs. (3)–(6), aggregation functions as in Eqs. (7) and (8), range and max-mean as in Eqs. (9) and (10), and anomaly detection as in Eq. (16), new features are created. These features are essential to improve the classification results.

Correlation coefficients are calculated on ${scaled\_X}^{anomaly}$ to identify the features that are most influencing the target. Considering that the initial datasets have about 20 numerical features on average, after calling $feature\_engineering$ procedure and $anomaly\_detection$ will lead to more than 200 numerical features. Therefore, $feature\_selection$ is an important procedure to reduce the dataset and retain the most significant features or reduced dataset ($red\_X$) that enhances the learning process of the ML algorithms.

17 $$featur{e}\_{selection\left({scale{d}_{X}}^{anomaly},y\right)}\to re{d}_{X}.$$

Then, the two datasets will be split into four datasets for training and testing the performance of the ML algorithms.

18 $$split\left(re{d}\_{X}, y, sampl{e}\_{size}\right)\to {X}_{train},{y}_{train}, {X}_{test},{y}_{test},$$

19 $$sampl{e}\_{size}\in \left[0.1{\div}0.3\right].$$

If the target distribution shows skewness, the unbalanced datasets ${X}_{train}$, ${y}_{train}$ are corrected with an $oversampling$ procedure, SMOTE or ADASYN, using a sampling strategy ratio that can vary from 0.1 to 1.

20 $$oversampling\left({X}_{train},{y}_{train},ratio\right)\to {X}_{train}^{b}, {y}_{train}^{b},$$

21 $$oversampling\in \left\{SMOTE, \,ADASYN\right\},$$

22 $$ratio \in \left[ {0.1{{ \div }}1} \right].$$

### Model selection, metrics and tunning

For training the classifiers using balanced datasets ${X}_{train}^{b},\, {y}_{train}^{b}$, we implement several ML algorithms and use the classifiers to generate ${y}_{predict}$:

23 $$train\left(classifier\left({X}_{train}^{b}, {y}_{train}^{b}\right)\right)\to classifier\left({X}_{test}\right)\to {y}_{predict}.$$

There is a high variety of classifiers. Some of the popular classifiers are mentioned below:

24 $$classifier\in \left\{Logistic\, Regression, \,Decision\, Tree, \,Random \,Forest\right\}.$$

By comparing the target with the predicted target or ${y}_{predict}$, several metrics are calculated. They represent an essential indicator to select the model.

Then, we create $df\_metrics$ to append $metrics$ for each classifier:

25 $$metric\in \left\{precision,\, recall, \,F1 \,score,\,AUC\, score, \,accuracy\right\}.$$

#### Specific metrics for classification

Precision is the positive predictive values (or true positive $tp$) showing ratio between hit and sum of false alarm (or false positive $fp$) and hit. It is what the model classifies as 1 or the suspicious consumers in our case.

26 $$Precision= \frac{tp}{tp+fp}.$$

Recall is also known as sensitivity of the model or Total Positive Rate ($TPR$) and shows the ratio between hits and the sum of missed (or false negative $fn$) and hits. False negative or miss shows the consumers that are fraudsters, but the model fails to identify.

27 $$Recall=TPR= \frac{tp}{tp+fn}.$$

Accuracy is probably the most important metric to assess the ML algorithms, but in classification problems with skewness, it is usually misleading.

28 $$Accuracy=\frac{tp+tn}{tp+tn+fp+fn}.$$

$tp$—true positive, also known as hit, meaning the fraudsters that the model correctly identifies as fraudsters; $tn$—true negative of correct rejection, meaning the non- fraudsters that the model correctly identifies as non-fraudsters; $fp$—false positive or false alarm, meaning the non- fraudsters that the model incorrectly classifies as fraudsters; $fn$—false negative or miss, meaning the fraudsters that the model incorrectly classifies as non-fraudsters.

As there is a permanent trade-off between $precision$ and $recall$, most of the times F1 score or harmonic mean of precision and sensitivity of the model is calculated.

29 $$F1 \,score=2\times \frac{precision \times recall}{precision + recall}.$$

AUC is the Area Under the Curve that is a function of $TPR$ defined above and False Positive Rate ($FPR$). It is a valuable metric in classification problems as it shows the capacity of the model to provide a fair classification. It varies between 0.5 and 1, 0.5 meaning a total failure of the classifier.

30 $$AUC=f\left(TPR, \,FPR\right),$$

31 $$FPR= \frac{fp}{fp+tn}.$$

Plotting learning curves allows us to estimate if the training datasets are sufficiently large to allow ML algorithms to learn. Usually, a score for training and testing are plotted considering a list of different training dataset sizes.

Validation curves are usually displayed to identify underfitting or overfitting problems of the model. They are investigated at length when the performance of the model is low. Furthermore, displaying ROC curves for different ML algorithms allow us to assess the performance of the model.

32 $$classifie{r}\_{plot}\left(lerning, validation, ROC\right)\to curves.$$

Tuning hyperparameters is usually done for the ML algorithms that perform best. For tuning, different strategies that will be described in the following section are available. Ranking the ML algorithms or model selection consists in sorting by one or more metrics in the data set that stores the metrics (for instance F1 score). Usually this is done inside of a procedure that checks the performance of a classifier with various hyperparameters.

33 $$sort\left(d{f}\_{metrics}\,by\, metric\right)\to bes{t}\_{{hyperpara{m}\_{classifier}}.}$$

#### Flowchart of the electricity consumption fraud detection

**Figure 2:**
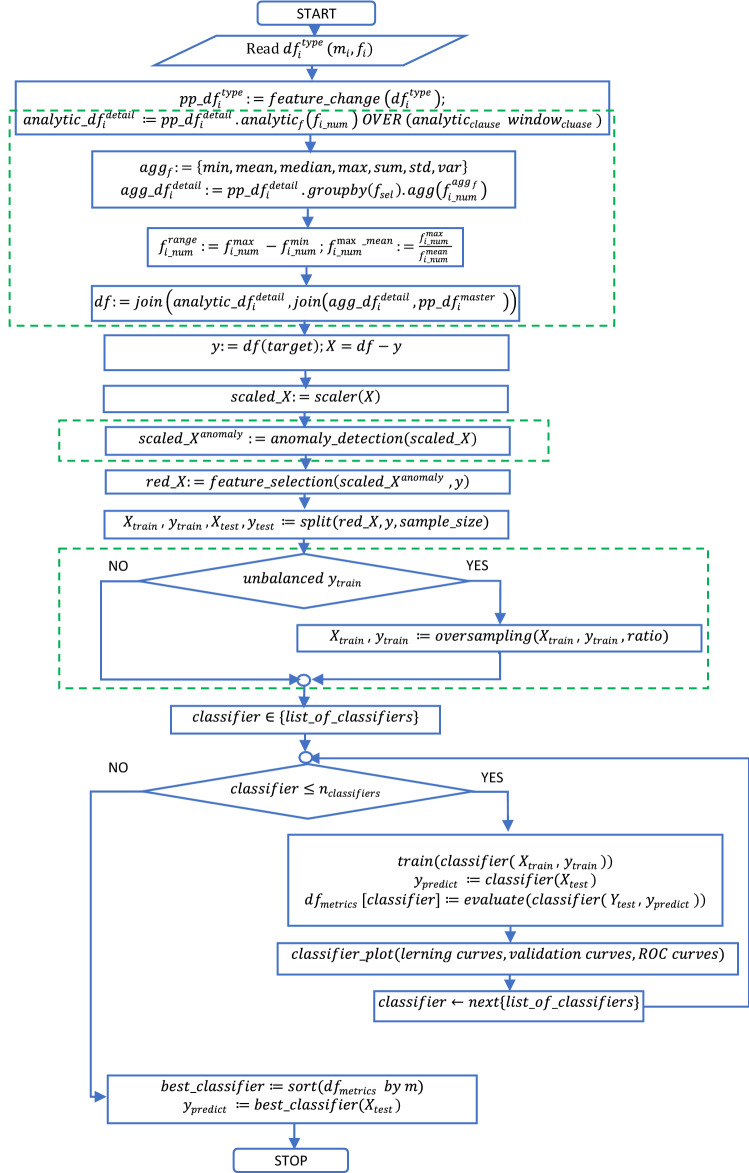
Flowchart of the electricity consumption fraud detection solution.

The flowchart of the electricity consumption fraud detection solution is presented in Fig. 2. The green marked areas represent the proposed procedure $feature\_engineering$ that is combined with anomaly detection to create new features and oversampling strategy to reduce the skewness of the model and improve the metrics.

Therefore, Fig. 2 provides more details and a better image in the fraud process detection. It represents the flowchart of the electricity consumption fraud detection solution and is based on the Eqs. (1)–(33). Figure 2 reflects the feature engineering procedure proposed in this paper, balancing the training data sets, oversampling or treating the skewness, training the models, calculating the metrics, and selecting the best algorithm for classification. It is significant as it provides details in implementing extensive feature engineering and integrating the steps of fraud detection with challenging data sets assuring the replicability of the proposed solution.

## Results

### Exploratory data analyses: data pre-processing and feature engineering

The datasets refer to the consumers from Tunisia (Africa) and their invoices, consumption, status of conventional meters and readings^37^. Two data sets as *.csv* files: *client* with 135,493 records and 6 features (*client_id, district, client_categ, region, creation_date, target*) referring to client information and *invoice* with 4,471,651 records and 16 features (*client_id, invoice_date, tarif_type, counter_number, counter_statue, counter_code, reading_remarque, counter_coefficient, consommation_level_1, consommation_level_2, consommation_level_3, consommation_level_4, old_index, new_index, months_number, counter_type*) referring to billing information are analyzed in the next paragraphs to understand the complexity of the input data^37^. The original 6-variable or feature definition of the client data set is: *client_id* is the unique id for client; *district* is the precise district location for each client; *client_categ* is the category client belongs to; *region* is the area where the client is located; *creation_date* is the date when the client was created (inserted into the database); *target* is the dependent variable or the fraud indication: fraud 1, not fraud 0. The original 16-variable or feature definition of the invoice data set contains billing information referring to: *client_id* is the unique id for client—this variable or feature allow us to join the two data sets; *invoice_date* is the issue date for each invoice; *tarif_type* indicates the type of tax; *counter_number* is the identifier of each counter or meter that belongs to a client; *counter_statue* takes up to five values indicating the status of the counter (such as: working fine, not working, on hold, etc.); *counter_code* is an additional identifier of the counter; *reading_remarque* is the note that an investigator from the utility company (grid operator, supplier) takes when reading the counter (if the counter is tampered, the investigator gives a remark); *counter_coefficient* is a coefficient that is provided when a standard consumption level is exceeded, *consommation_level_1, consommation_level_2, consommation_level_3, consommation_level4* are indicating four consumption levels, *old_index* and *new_index* provide the previous and current consumption values, the difference between the new and old indexes provide the consumption that is invoiced; *months_number* represents the number of months between readings; *counter_type* indicates the type of the counter that belongs to a client. The target (fraud 1, not fraud 0) is a dependent variable that belongs to client data set and depends on the consumption reading and invoice characteristics that are part of the invoice data set. Therefore, it is essential to join the two data sets (client and invoice) and analyze the irregularities that stem from data to be able to identify potential fraudster.

In the first scenario, we analyzed the unique values of each column, their types, outliers, and the distribution of features. To combine the two datasets, a merge procedure in Pandas Python library was implemented. The merged dataset has 4,476,749 million of rows and 21 columns. Usual feature engineering is performed for both scenarios executing the procedure $feature\_change$. It consists in transforming strings into dates, extracting month, year, weekdays and weekends from dates—for *creation_date* and *invoice_date* features, encoding and recoding categorical variables, computations such as monthly, daily consumption, delta index, delta time, removing redundant variables after computation, etc.

**Figure 3:**
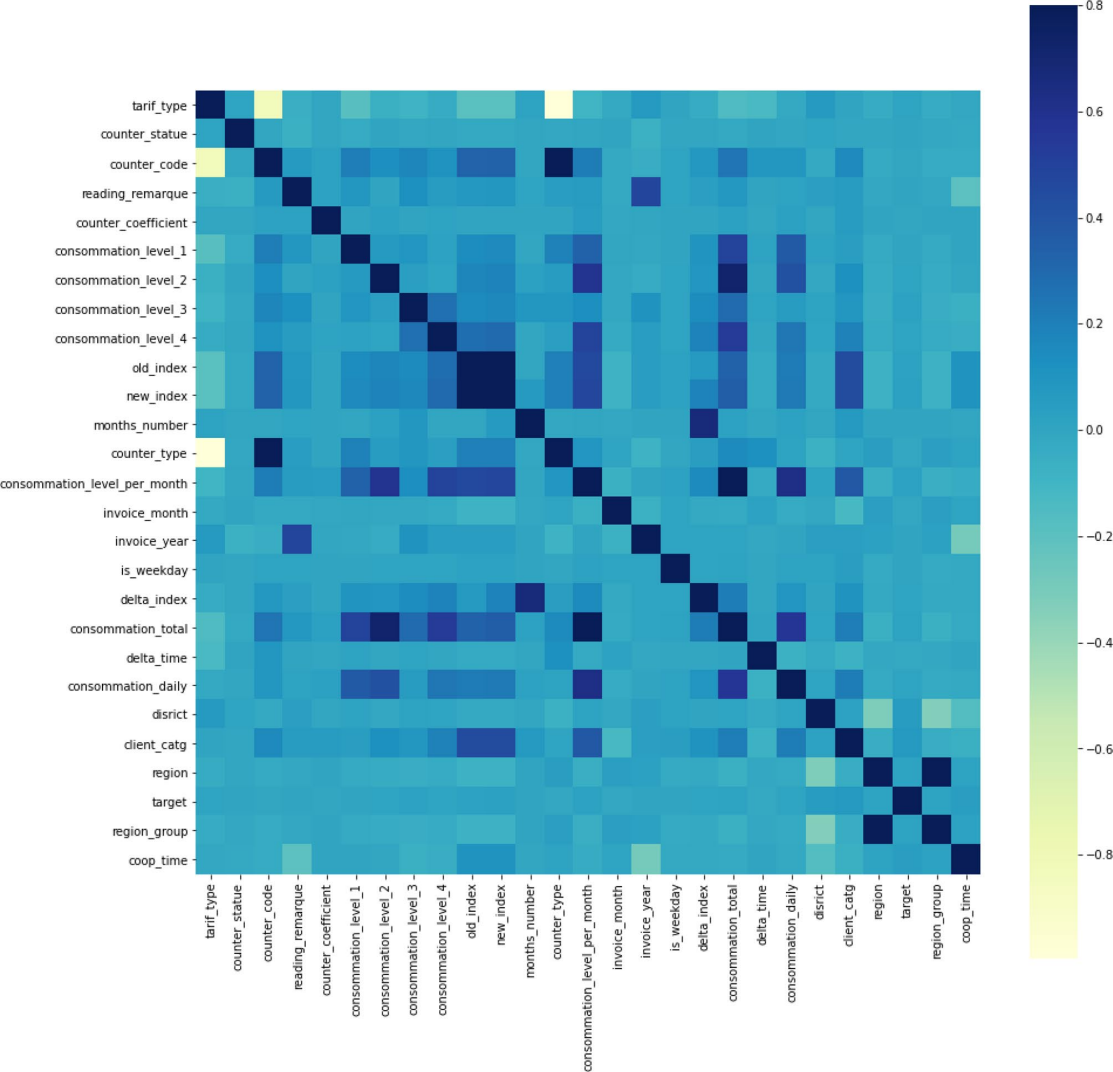
Correlation matrix on merged dataset.

Many machine learning algorithms (such as: linear and logistic regression, nearest neighbours, neural networks, SVM with radial basis kernel functions, principal component analysis, and linear discriminant analysis) perform better or converge faster when the values are on a relatively similar scale. *MinMaxScaler* and *StandardScaler* from sklearn Python library should be the default transformations as they do not distort the values, but *RobustScaler* is much better when there are numerous outliers. Furthermore, *Normalizer* normalizes records, not feature columns as other scalers, with *l2* or *l1* normalization^38^. Pearson correlation coefficients are calculated to estimate the dependency between the target and the other variables. From Fig. 3, we can conclude that the variables (features) are relatively weakly correlated with the target.

Thus, in the first scenario, we implement the methodology described in “Methodology” section except for the procedure $feature\_engineering$ for aggregating the features, double merging the datasets and combining anomaly detection with supervised ML algorithms. In the second scenario, we added the procedure $feature\_engineering$ according to the methodology and compare the results of the two scenarios, underlying the importance of extensive feature engineering and the combination of supervised and unsupervised ML algorithms. After calling the above-mentioned procedure, almost 200 new features are created. Thus, the dataset in the second scenario is considerable, with over 4 million rows and over 200 columns. Several examples of feature engineering with SQL analytic functions are provided in Table 2.

**Table 2:** Example of feature engineering with SQL analytic functions.

Feature	Feature engineering
f1_con_level1_avg Average consumption in the same invoice period of consumers with similar tariff type	SELECT tarif_type, client_id, invoice_date, consommation_level_1 AVG(consommation_level_1) OVER (PARTITION BY tarif_type ORDER BY invoice_date ROWS BETWEEN 1 PRECEDING AND 1 FOLLOWING) AS f1_con_level1_avg FROM invoice ORDER BY tarif_type, invoice_date
f2_con_level1_avg Average consumption of consumers with the same tariff that were invoiced before the current one	SELECT tarif_type, client_id, invoice_date, consommation_level_1 AVG(consommation_level_1) OVER (PARTITION BY tarif_type ORDER BY invoice_date ROWS BETWEEN unbounded PRECEDING AND current row) AS f2_con_level1_avg FROM invoice ORDER BY tarif_type, invoice_date;
f3_con_level1_avg Average consumption of consumers with the same tariff that were invoiced after the current one	SELECT tarif_type, client_id, invoice_date, consommation_level_1 AVG(consommation_level_1) OVER (PARTITION BY tarif_type ORDER BY invoice_date ROWS BETWEEN current row AND unbounded following) AS f3_con_level1_avg FROM invoice ORDER BY tarif_type, invoice_date
f4_con_level1_avg Average consumption of consumers with the same tariff but with difference of consumption of ± 100 to the current one	SELECT tarif_type, client_id, invoice_date, consommation_level_1 AVG(consommation_level_1) OVER (PARTITION BY tarif_type ORDER BY consommation_level_1 range BETWEEN 100 preceding AND 100 following) AS f4_con_level1_avg FROM invoice ORDER BY tarif_type, consommation_level_1
f5_con_level1_min f6_con_level1_max Minimum and maximum consumption of the consumers with the same tariff with consumptions less or equal to the current one	SELECT tarif_type, client_id, invoice_date, consommation_level_1 MIN(consommation_level_1) OVER (PARTITION BY tarif_type ORDER BY consommation_level_1 rows BETWEEN unbounded preceding AND current row) AS f5_con_level1_min MAX(consommation_level_1) OVER (PARTITION BY tarif_type ORDER BY consommation_level_1 rows BETWEEN unbounded preceding AND current row) AS f6_con_level1_max FROM invoice ORDER BY tarif_type, consommation_level_1

Such features capture the irregularities from data of consumers with similar tariff for instance enhancing the classification process and fraud detection.

Considering the large data set, the best features were selected using a feature selection procedure according to the Fisher score for both scenarios. Selecting the right set of features or reducing dimensionality for classification is significant to improve the performance of ML algorithms^39–41^. Thus, computing and using feature importance scores to select the best features is an important stage that has to be performed before running the algorithms. Fisher score is typically used in binary classification problems and is defined as ratio between the distance between the sample means for each class (1 or 0) per feature and their variances. The analyzed dataset is highly unbalanced as the ratio is 12:1. SMOTE^42^ and ADASYN^43,44^ are applied to the training datasets. However, considering the large training dataset, oversampling considerably increases the computational time. An issue with imbalanced classification is that there are too few cases of the minority class (1—because usually the fraudsters are a minority compared with the rest of electricity consumers or majority) for a model to effectively learn the decision boundary that is essential in classification problems^45,46^. SMOTE synthesizes new samples from the minority class, whereas ADASYN is an extension of SMOTE that generates synthetic samples inversely proportional to the density in class 1 or minority class. There are other extensions of SMOTE, such as Borderline-SMOTE^47^ that is more selective about the sample in the minority class that are oversampled. This approach oversamples those difficult samples on the borderline that tend to be misclassified.

The differences between the two scenarios are: (a) adding in the second scenario SQL analytic functions to identify the spikes in current/previous consumption; (b) grouping and aggregating the data by applying several statistical functions such as *min*, *mean*, *max*, *std*, *sum*, *var*, *median*; (c) combined with anomaly detection that generates a valuable new feature that better reveals whether the sample is normal or not.

### Metrics evaluation and parameters of the selected models

Ten supervised algorithms for classification, also known as classifiers: *Logistic Regression* (LR), *Stochastic Gradient Descending* (SGD), *eXtreme Gradient Boosting* (XGB), *Decision Tree* (DT), *Random Forest* (RF), *Multi-Layer Perceptron* (MLP), *Light Gradient Boosting* (LGB), *Quadratic Discriminant Analysis* (QDA), *CatBoosting* (CB) and *AdaBoosting* (AB) are selected for the first scenario. Whereas, for the second scenario, the following supervised algorithms are implemented: LR, XGB, DT and RF and an unsupervised algorithm for anomaly detection: *IsolationForest* is also implemented to create a new feature for the dataset.

Before tuning, one has to know the significance of the hyperparameters and their inter-dependency. Usually, this stage requires more experience and understanding of the trade-off between performance and computational time and resources. Tuning the hyperparameters with *GridSearchCV*, *Optuna* or other tuning tools is time-consuming and sometimes fails to return results, especially when the datasets are large^48,49^. Thus, identifying the most important hyperparameters for a specific algorithm and performing several loops to find the best combination of the hyperparameters from several metrics point of view is probably the best solution. The results could be stored into a *dataframe* that can be sorted to obtain the desired results. Sometimes, it is better to check each significant hyperparameter and plot validation curves to identify under- or overfitting problems of the model^50^. Depending on the problem and dataset characteristics, precision or recall optimization is possible. Also changing the threshold can fine-tune the results^51^.

#### Metrics evaluation

**Figure 4:**
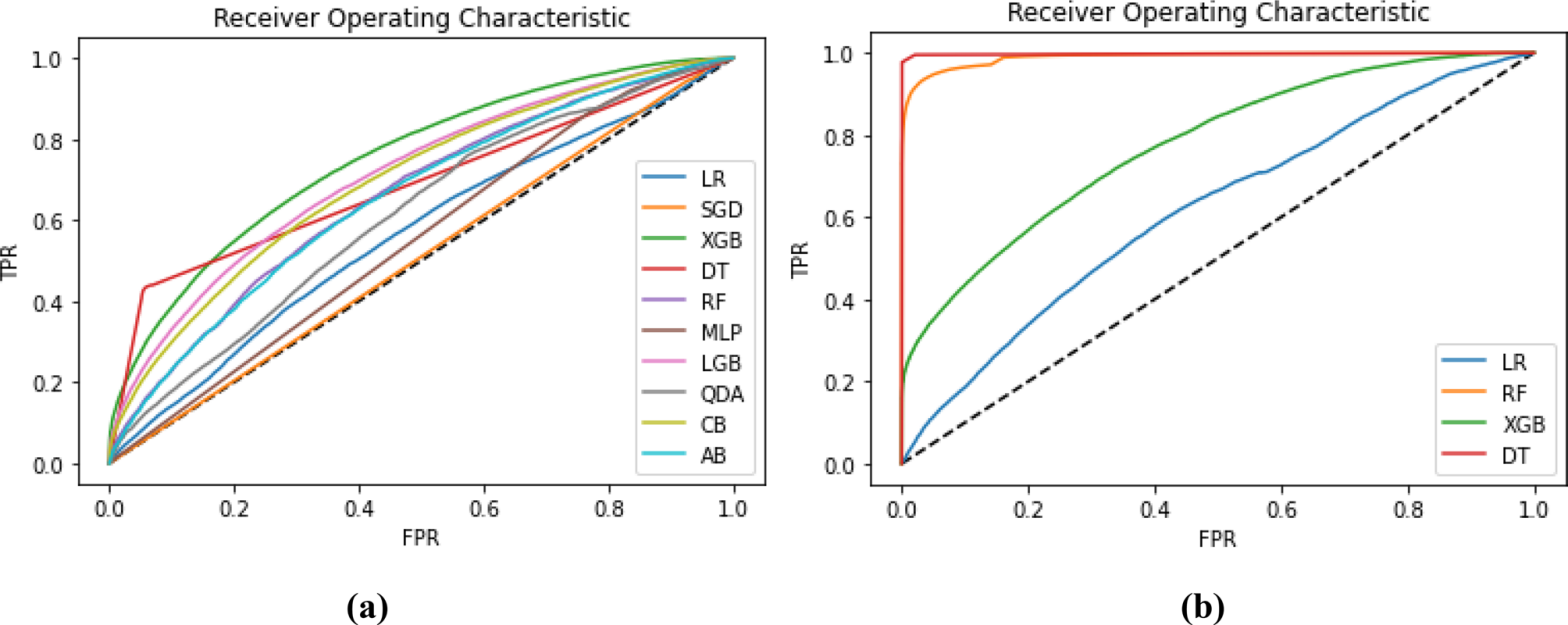
ROC curves—scenario 1 (**a**), scenario 2 (**b**).

Usually, the first metric investigated is the accuracy or performance of the model. In some cases, the error is also a good indicator. However, high accuracy not always indicates the best solution (algorithm) because in classification problems, precision and recall are more important. Precision depends on the *false positive*, whereas recall depends on the *false negative*. Thus, there is a trade-off between the two metrics, whereas F1 score or F score takes both precision and recall into account. Therefore, F-score better reflects the performance of the model. Furthermore, AUC score and ROC curves show the supremacy of the algorithm since they represent the capacity of the model to classify samples. Using learning and validation curves is also important when tuning the model^52^. In the first scenario, ten ROC curves are drawn in Fig. 4 (left). The best performant algorithm is DT, followed by XGB. Whereas in the second scenario, we run four algorithms, out of them RF and DT are the best classifiers. We may also notice that RL, SGD and MLP completely failed to classify samples. Furthermore, we implemented Support Vector Classifier (SVC), but with the large data set, it did not run properly.

**Figure 5:**
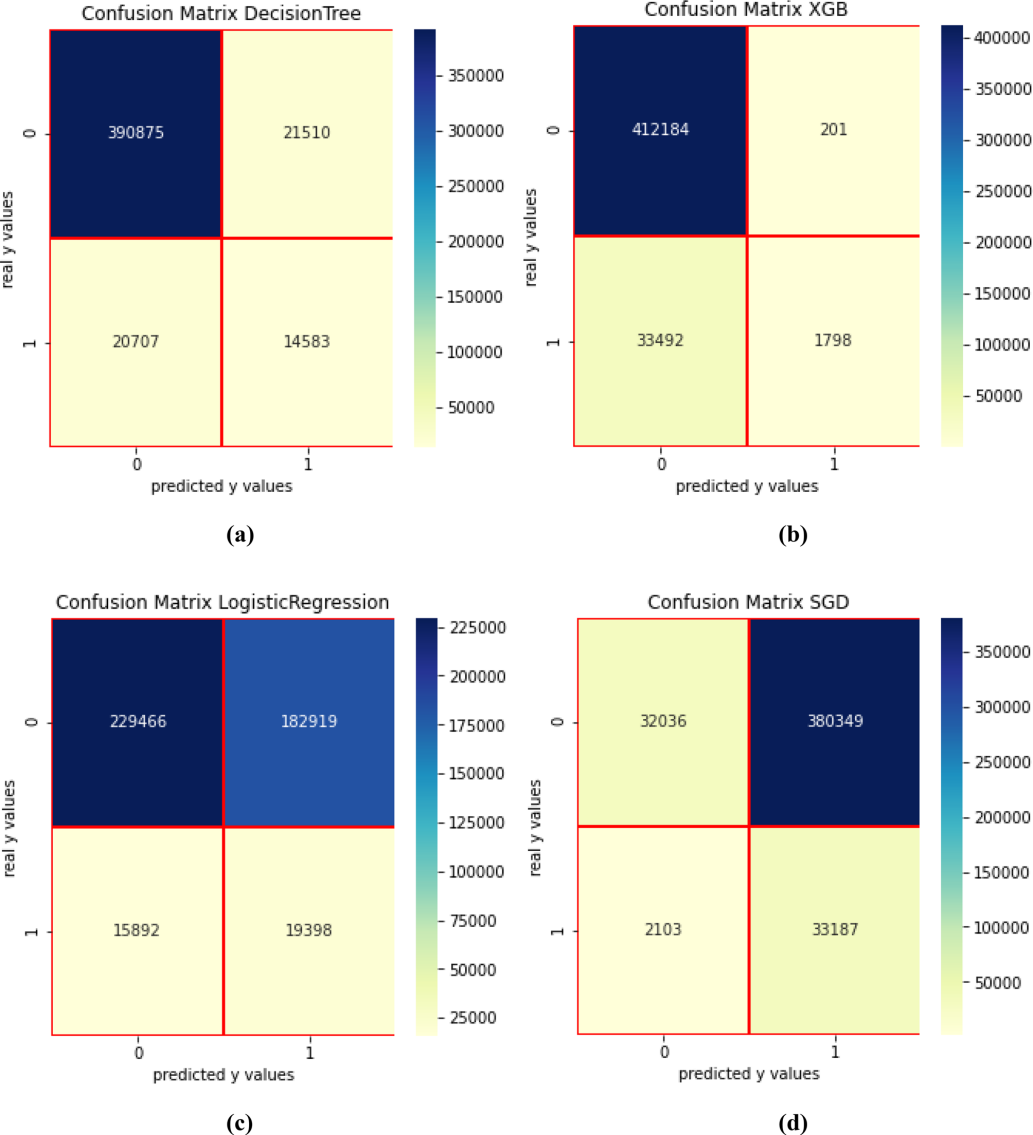
Confusion matrix—scenario 1. (**a**) DT, (**b**) XGB, (**c**) LR, (**d**) SGD.

For the second scenario, four ROC curves are shown as in Fig. 4 (right). The best performing algorithm is DT, immediately followed by RF. Confusion matrixes for scenario 1 for DT, XGB, LR and SGD are presented in Fig. 5.

Over 41.32% of the fraudsters were identified with DT, whereas only 5.22% represent *false positive* or consumers that are identified as fraudsters, but they are not. As for XGB, only 5.02% of the fraudsters are identified and 0.05% represent *false positive*. SGD identified over 94.04% of the fraudsters, but the *false positive* is also very high (92.23%). On the other side, LR identified more than 54.97% of the fraudsters, but the *false positive* is also high (44.35%). The classification report for DT is presented in Table 3.

**Table 3:** Metrics for the first scenario.

Classifier	Target	Precision	Recall	F1 score	Support	Accuracy	AUC score
DT	0.0	0.95	0.95	0.95	412,385	0.91	0.6805
	1.0	0.40	0.41	0.41	35,290		

The data was scaled with *MinMax* scaler and AUC score slightly increased from 0.6805 to 0.6812, whereas the other scalers did not improve the results. Increasing the number of features from 9 to 15, the AUC score also increased to 0.6993. Applying SMOTE with sampling strategy 0.5 slightly increased AUC score from to 0.7281, and when the sampling strategy is increased from 0.5 to 1 further increased AUC score to 0.7331. ADASYN did not improve the results. Furthermore, in the first scenario, the model was not sensitive to the newly added anomaly detection feature.

For the second scenario, the data pre-processing steps were similar to the previous scenario. However, new features are created with SQL analytic functions in the *invoice* dataset*.* Before first merging, the *invoice* dataset is grouped by *client_id* and *counter_type* features and *min*, *mean*, *sum*, *std* aggregate functions are calculated. Furthermore, the reduced *invoice* dataset is merged with the *client* dataset by *client_id* and the result is merged with detailed invoices data to which new features were added with SQL analytic functions, so that the aggregated and detailed data to be included in the same dataset. The purpose of this double merging is to apply an anomaly detection algorithm, create a new valuable feature and identify anomalies between aggregated values and details at the invoice level. The number of features before selecting the best 15 for training was over 200. Furthermore, we introduce a new feature computed with a powerful unsupervised algorithm for anomaly detection—Isolation Forest. In the first scenario, it did not improve the performance of the algorithm because it could not detect anomalies in the consumption data. However, the SQL analytic functions and double merging the datasets allowed the comparison in the invoice data between the aggregated values and previous invoices as well as between the current and previous consumption over sliding windows using partitions as described in “Methodology” section.

**Figure 6:**
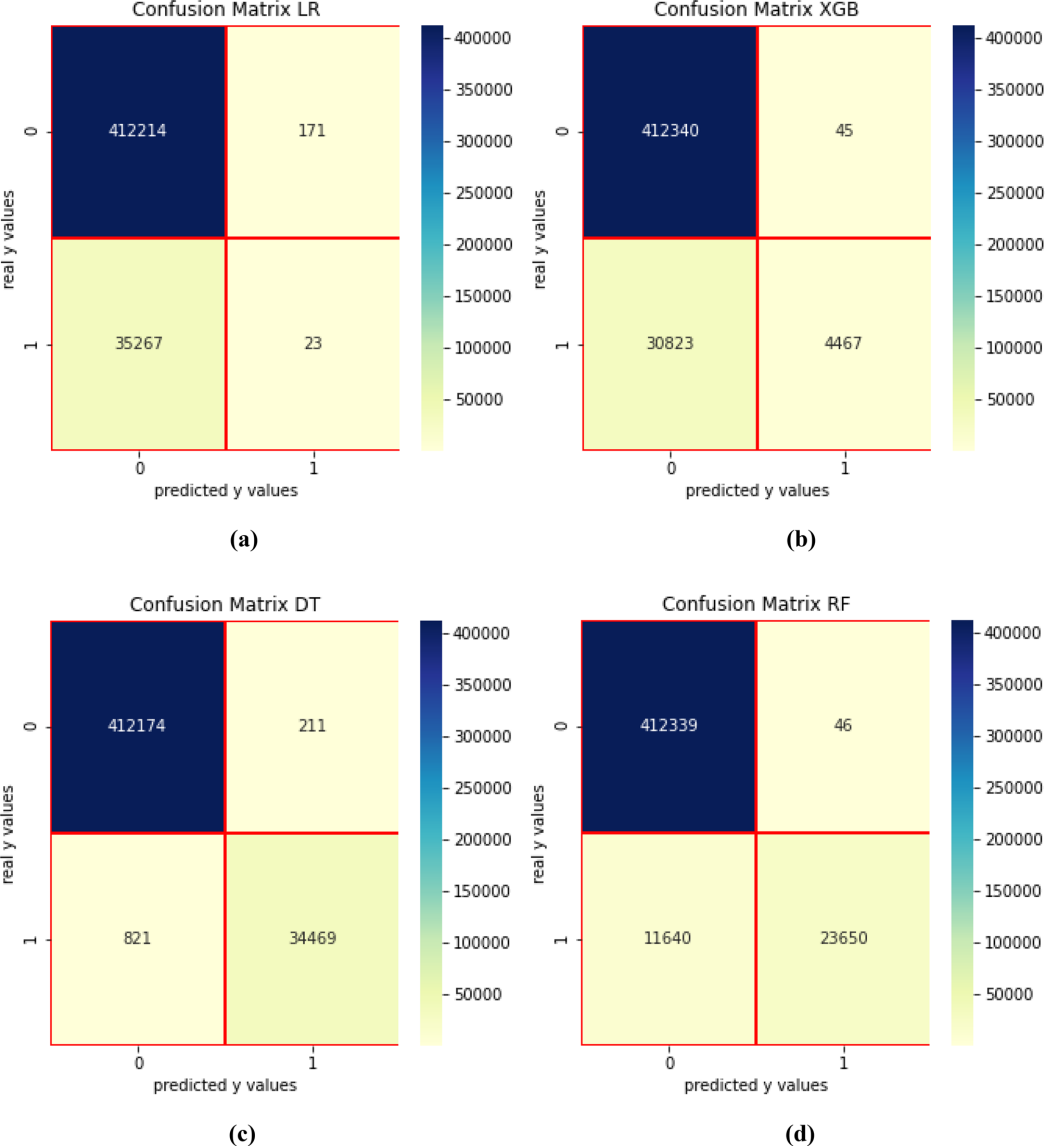
Confusion matrix—scenario 2. (**a**) LR, (**b**) XGB, (**c**) DT, (**d**) RF.

**Figure 7:**
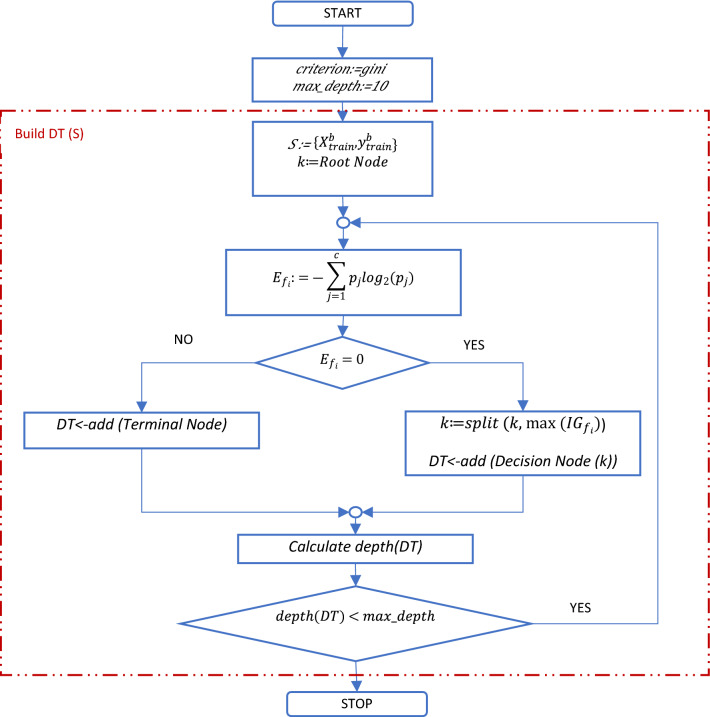
DT training process.

**Figure 8:**
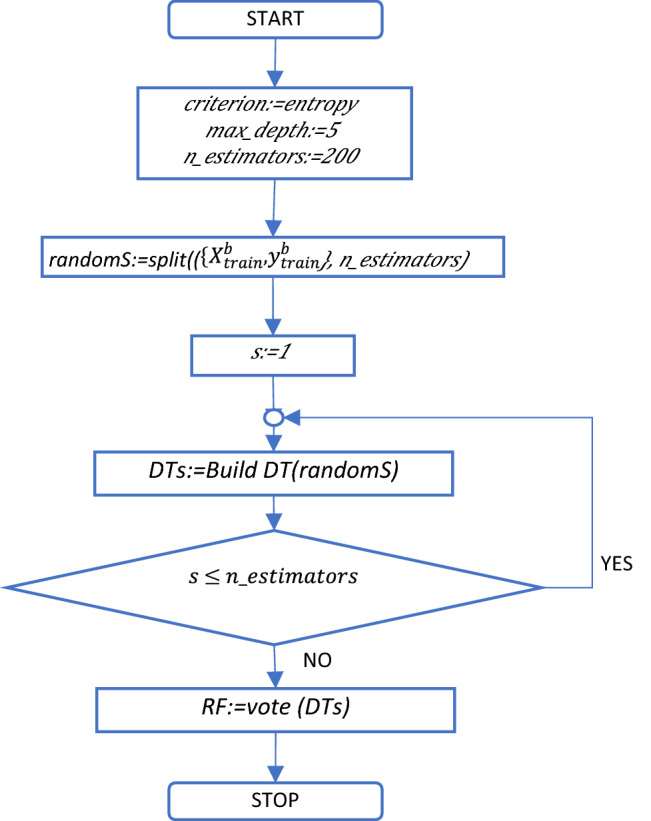
RF training process.

The confusion matrixes in Fig. 6 show the performance of the four ML algorithms implemented in the second scenario. LR and XGB performances are better in comparison with the first scenario, but the best results are obtained with DT and RF algorithms. An overview on the DT and RF algorithms is given in Figs. 7 and 8.

For RF, the *false positive* is only 46 which is an outstanding performance since a very small number of consumers was incorrectly identified as suspicious. However, out of 35,370 consumers, 11,640 were missed by this algorithm. Therefore, RF identified 67.01% of the problematic consumers and the *false positive* is just 0.011%. Furthermore, DT provides excellent results. Over 97.67% of the fraudsters were identified with DT, whereas only 0.05% represent *false positive* or consumers that are identified as fraudsters, but they are not. Significant metrics for the second scenario are given in Table 4.

**Table 4:** Metrics for the second scenario.

No.	Classifier	Target	Precision	Recall	F1 score	Support	Accuracy	AUC
1	DT	0	1	1	0.99	412,385	0.997695	0.997
		1	0.99	0.98		35,290		
2	RF	0	0.97	1	0.89	412,385	0.973896	0.990
		1	1	0.67		35,290		
3	XGB	0	0.93	1	0.59	412,385	0.931048	0.776
		1	0.99	0.13		35,290		
4	LR	0	0.95	0.63	0.48	412,385	0.920840	0.616
		1	0.12	0.62		35,290		

#### Parameters of the DT and RF algorithms

DT are straightforward, non-parametric models based on *if-then-else* decision rules that are built on the training set. DT are composed of a root node that contains the initial training set, decision nodes that contain the decision rules and terminal nodes or leaves with the final classification results. Thus, the DT learning algorithm splits the input set (${X}_{train}^{b}$) into sub-sets until it forms the final leaves of the tree or terminal nodes to predict the target (${y}_{train}^{b}$). First, the root node is built using the entire training set and is divided iteratively into sub-sets (*k*) using a splitting criterion that uses entropy or Gini index as cost functions to measure the quality of the split made by different features. Entropy is calculated for each feature (${f}_{i}$) with Eq. (34) and it is associated with the randomness or uncertainty of the processed information.

34 $${E}_{{f}_{i}}=-\sum_{j=1}^{c}{p}_{j}{log}_{2}\left({p}_{j}\right),$$

where *c* represents the classes of the target variable (0,1); ${p}_{j}$—the proportion of the samples that belongs to class *c* for a particular node. A higher value of entropy in a decision node leads to further splitting of the input in that node. A node with a zero value represents a leaf node. Gini index is similar with entropy, and it is calculated using Eq. (35):

35 $${G}_{{f}_{i}}=1-\sum_{j=1}^{c}{\left({p}_{j}\right)}^{2}.$$

Building the decision rules of the DT, the information gain or IG is calculated for each feature to compare the entropy before and after the split into *k* subsets using Eq. (36):

36 $${IG}_{{f}_{i}}={E}_{{f}_{i}}-\sum_{k=1}^{K}{E}_{{f}_{i}}\left(k\right).$$

For further splitting, at each iteration the DT algorithm selects the feature that has the maximum $IG$.

In simulations, we used entropy as the splitting criterion and, to avoid overfitting, a maximum depth of the DT is considered using the hyperparameter *max_depth* set to 10. The value is determined in the tuning process.

Since the DT represents a straightforward model, the overfitting issues are usually encountered. To overcome this issue, RF introduces a more sophisticated model that combines multiple DTs to obtain a better classification of the target variable. Thus, RF is composed of an ensemble of trees that uses the bagging technique to randomly extract subsets of features for the splitting process. Then, the trees are trained and build on the randomized samples. The predictions of each tree are combined using the voting method to provide the result of the classification. In case of RF, the same measures are used to determine the split. Regarding the tuning of the hyperparameters used in simulations, *max_depth* is set to 5 and the number of trees or estimators (*n_estimators*) to 200. Figure 7 shows the flowchart of the building process (training) of the DT and Fig. 8 the training of the RF on the $({{X}_{train}^{b}}, \,{{y}_{train}^{b}})$ dataset.

Balancing the data with SMOTE or ADASYN did not increase the performance of the classification and the computation time increased due to the larger number of rows synthetically created to balance the 1 values of the target. However, such balancing methods should be reasonably implemented, not as in Ref.^53^ where both training and testing datasets with similar fraud detection were balanced. If the test dataset is altered whatsoever, we risk obtaining an algorithm that overall detects more fake or synthetically created fraudsters than real ones.

## Conclusion

In this paper, we investigated over 135 thousand of electricity consumers with conventional meters from Tunisia and their invoices stored in large datasets with over 4 million of records and identified the most suspicious, combining two types of machine learning algorithms: anomaly detection—unsupervised and classifiers—supervised algorithms.

To enhance the results of classification process with large unbalanced and uncorrelated real datasets, we proposed an extensive feature engineering solution that consists of implementing SQL analytical functions that identify the differences between one invoice and the previous/following ones, grouping and aggregating data using statistical functions, implementing both supervised classifiers and anomaly detection.

By comparison with the case without extensive feature engineering, the performance of the model significantly increases. Classifiers such as Random Forest and Decision Tree performed much better than in the first scenario without feature engineering. Furthermore, eXtreme Gradient Boosting can be reliable especially when the grid utility has limited resources to further investigate the suspicious consumers.

Several policy implications can be extracted from our study:

- Machine learning algorithms are capable of providing a list of suspicious consumers. Thus, on-site crews will concentrate only upon a group of consumers that are prone to theft reducing the human resource training and mobility related cost of the utility company.
- Further investigations will definitely be required to *confirm* that problematic consumers are involved in fraudulent activities, thus a performant fraud detection solution is a salient tool for utility companies that face Non-Technical Losses (NTL).
- Decision regarding choosing an algorithm depends on the utility company’s budget as each set of results leads to periodic on-site inspection costs and revenue generation from fines and fraud recovery.
- Continuous monitoring of consumption is required so that fraud is quickly detected and investigated as this proof is important at the inspection stage.
- Fraud in electricity consumption should be discouraged by large fines and other measures similar to criminal activities to minimize the NTL that are in the end paid by entire society.
- Burdensome fines and criminal record will reduce the electricity price by decreasing the NTL component.
- Training and monitoring the crews’ activities, especially in countries where corruption is high, are essential in fighting the fraud in electricity consumption.

As a limitation, the proposed approach can only be replicated for similar datasets measured by conventional meters. However, the SQL analytic function are adequate for smart meter-recorded data as well. For this data, there are other approaches that involve specific machine leaning algorithms and time series feature extraction library for electricity consumption fraud detection in smart grids.

However, although the trend is to gradually replace the conventional meters, there are still large regions even in the developed countries that continue to measure electricity with conventional meters. So, the conventional meters are far from being replaced in the next 10 years all over the world, both meter types (conventional and smart) will coexist for a longer period and will require performant and practical methods to detect frauds.

We noticed that out of the 30 references, from “Literature review” section, that carried out comprehensive electricity consumption fraud detection, only 5 are related to the conventional meter-recorded data and only 7 approaches feature engineering.

Future work will focus upon detecting fraud in smart meter readings since the number of smart metering rollout projects is increasing and suspicious or fraudulent consumers will generate more spikes in their consumption pattern as smart meter readings are more frequent.

## Data Availability

We state that the data is open and available here: https://zindi.africa/competitions/ai-hack-tunisia-4-predictive-analytics-challenge-1/data.
